# Supplementary material for: Multivalent Epigraph Hemagglutinin Vaccine Protects against Influenza B Virus in Mice
Source: Pathogens. 2024 Jan 23;13(2):97. doi: 10.3390/pathogens13020097 (PMC10892733; doi:10.3390/pathogens13020097)
Supplement: Supplementary file 1 [file pathogens-13-00097-s001.zip › pathogens-2815766-supplementary.pdf]

A

Ad-HA	VP/mL	IFU/mL	VP:IFU Ratio
Vic-Epi 1	1.26e12	2.27e10	56:1
Vic-Epi 2	7.95e11	4.13e9	192:1
Vic-Epi 3	1.18e12	5.59e9	211:1
Yam-Epi 1	1.12e12	4.08e9	274:1
Yam-Epi 2	6.25e11	1.58e9	395:1
Yam-Epi 3	7.55e11	3.48e9	217:1

B

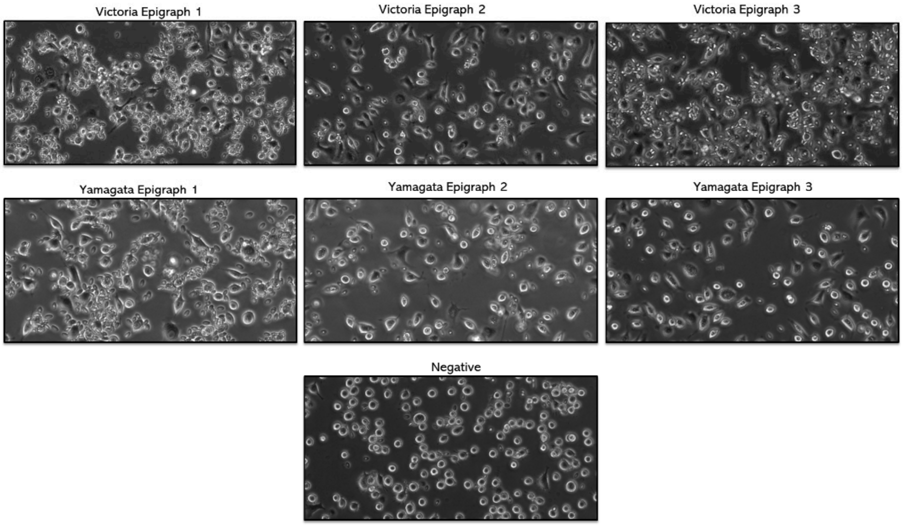

C

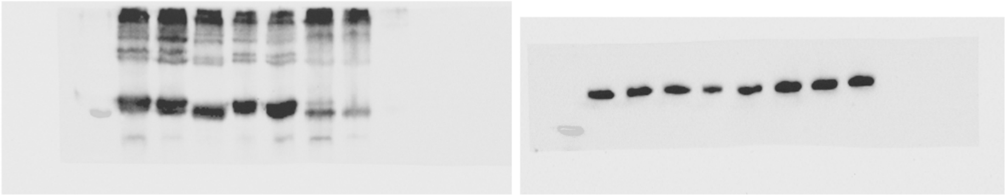

Supplemental Figure 1. Characterization of IBV Epigraph HA proteins. (A) Infectious unit to viral particle ratio determined by AdenoX Rapid Titer. (B) Rosette Analysis for Epigraph HA proteins. (C) Full western blot analysis of Epigraph HA proteins (Lane 1: ladder; lane 2: Victoria Epigraph 1; lane 3: Victoria Epigraph 2; lane 4: Victoria Epigraph 3; lane 5: mis-pipette; lane 6: Yamagata Epigraph 1; lane 7: Yamagata Epigraph 2; lane 8: Yamagata Epigraph 3; lane 9: uninfected control).

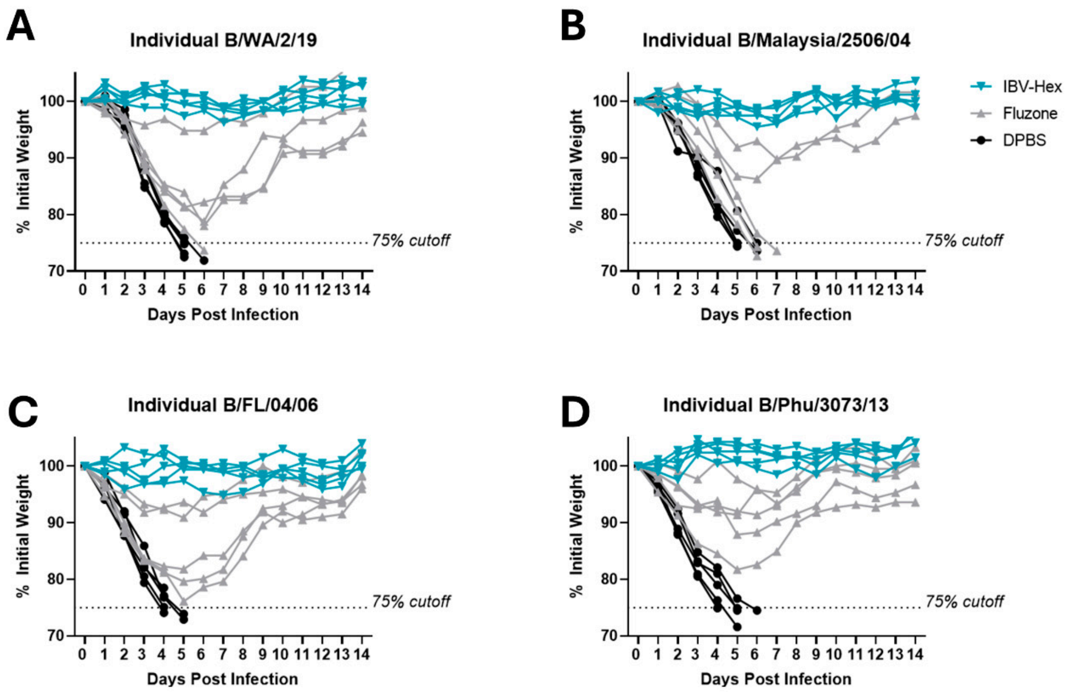

Supplemental Figure 2. Weight loss of individual mice after lethal challenge. Mice were challenged with 100MLD$_{50}$ of Victoria-like IBV (A) B/WA/2/19 and (B) B/Malaysia/2506/04 or Yamagata-like IBV (C) B/FL/04/06 and (D) B/Phu/3073/2013.
